# Symptom Perception and Influencing Factors in Chinese Patients with Heart Failure: A Preliminary Exploration

**DOI:** 10.3390/ijerph17082692

**Published:** 2020-04-14

**Authors:** Hong Luo, Deborah F. Lindell, Corrine Y. Jurgens, Yongsheng Fan, Liping Yu

**Affiliations:** 1 Department of Nursing, School of Health Sciences, Wuhan University, Wuhan 430071, China; luohong1020@whu.edu.cn (H.L.);; 2 Frances Payne Bolton School of Nursing, Case Western Reserve University, Cleveland, OH 44106, USA; 3 William F. Connell School of Nursing, Boston College, Boston, MA 02647, USA; 4 Department of Public Health and Preventive Medicine, School of Health Sciences, Wuhan University, Wuhan 430071, China

**Keywords:** heart failure, symptom perception, self-care, self-management

## Abstract

A growing body of evidence supports the fact that optimal health-related quality of life is largely dependent on patient competence in symptom perception. However, many studies have reported poor symptom perception in patients with heart failure. In China, there has been no previous research on assessing the symptom perception ability of patients with heart failure. This study aimed to describe how Chinese patients with heart failure perceive their symptoms, as well as to explore their influencing factors. A theory-based, descriptive, correlational cross-sectional design was used in this study. Data on symptom perception and factors related to symptom perception were collected via structured interviews and medical records. A convenience sample of 208 hospitalized patients was enrolled. The degree of symptom perception in this study was at a high level. The results showed that the level of depression, the New York Heart Association functional class, the left ventricular ejection fraction, and educational background were identified as independent factors of symptom perception in Chinese patients with heart failure. The degree of symptom perception of patients with heart failure was affected by personal, psychological, and physiological factors. Health policy and healthcare providers should pay more attention and deepen the understanding to Chinese patients with heart failure to provide better healthcare.

## 1. Introduction

Heart failure (HF) has become a global disease, with an increasing prevalence of about 26 million people [1]. Projections show that the prevalence of HF would increase 46% from 2012 to 2030 in the United States, resulting in more than 8 million HF patients over 18 years of age [2]. HF has high hospitalization and readmission rates [3,4,5]. In China, the hospitalization rate of HF accounted for 20% of cardiovascular diseases, whereas the mortality rate of HF accounted for 40% [6].

More than 50% of HF patients were found to be re-hospitalized with HF-related symptoms within six months after discharge [7]. Symptom perception was considered as the most critical step to successful self-care management, as poor symptom perception could cause exacerbation of the HF and delays in treatment [8,9,10]. Among multiple definitions of symptom perception, Riegel defined *symptom perception* as including both symptom monitoring and symptom recognition, as well as body listening, symptom interpretation, and labeling [10,11]. 

HF patients are always under great pressure in managing their symptoms [12]. Patients with HF often cannot recognize their early symptoms very well, and they are either used to having symptoms or are unaware that their symptoms are getting worse [13]. Contributing factors might include an age-related declined function status, comorbidity, living with others, educational level, uncertainty about symptoms’ meanings, less vigilance regarding symptoms changes, and symptom duration [3,13,14,15]. 

Some research studies have described factors influencing symptom perception in the heart failure field [3,16]. However, to our knowledge, there has been no previous research focused on assessing the symptom perception ability of patients with heart failure in China. The twofold purpose of this study was to describe (1) how Chinese patients with heart failure perceive and respond to their symptoms, and (2) whether there is a relationship between the degree of symptom perception and the patients’ levels of anxiety, depression, the New York Heart Association (NYHA) functional class, left ventricular ejection fraction (LVEF), N-terminal pro B-type natriuretic peptide (NT-proBNP), comorbidity status, and duration of symptoms. 

## 2. Theoretical Framework

The theoretical framework for the study was the Theory of Heart Failure Self-Care (revised and updated) [10]. The theory provided a more specific and less abstract theory to use in efforts to describe the process that patients use to perform self-care [17]. In the theory, the self-care process was defined as a naturalistic decision-making (NDM) process, constructed by *self-care maintenance*, *symptom perception*, and *self-care management* [10]. Self-care referred to a linear process proceeding from self-maintenance, to symptom perception, to self-management [10]. 

The theory also indicated that situational characteristics (person, problem, and environmental factors) and self-efficacy might make the self-care process unique to each HF patients. Contributing factors influencing decision about the implementation of the whole self-care process include the individual’s experiences, knowledge, skill, and values [10]. Experience and knowledge affect self-management by helping to identify symptoms in situations and match to decisions and actions. 

Two propositions of the theory were outlined in this framework: (a) symptom perception was required for successful self-care management, and (b) decisions about self-care might be conscious or subconscious; these decisions reflect choices driven by the interactions of the person, problem, and environmental factors. Self-care management was measured by the duration of symptoms, which represented patients’ care-seeking behaviors in this study.

## 3. Methods

### 3.1. Design

A descriptive, correlational cross-sectional design was used. The study was performed from May 1, 2019 to October 31, 2019. Patients diagnosed with acute heart failure were consecutively recruited from five nursing units in three departments of cardiology of three university-affiliated hospitals in Wuhan, China. This study was approved by the Ethics Committee of the School of Medicine, Wuhan University (2019YF2040). A written informed consent was obtained from each participant before they participated in the study.

### 3.2. Sample

This study used a convenience sample approach to recruitment. A prior power analysis was used to determine proper sample size for correlation and regression analysis. By using G*Power 3.1.9.2 computer program software (Program written by Franz Faul, Univerität Kiel, Germany), based on a power of 0.80, an effect size of 0.30, and an alpha level of 0.05, the estimated sample size for correlation was 82. Meanwhile, on the basis of 10–20 participants per variable in the multivariate regression model, together with the ability to meet the aims, the minimum sample size for regression analysis was 180. Considering the heterogeneity of the population, 15% (27) was oversampled, and the estimated minimum sample size for in this study was 207 participants.

We included hospitalized patients admitted to the hospital due to a deterioration of heart failure. The inclusion criteria were patients (1) diagnosed with acute heart failure (not associated with a reversible cause); (2) over 18 years of age; (3) within 72 h of admission; (4) alert and able to understand the study, and to provide written informed consent; (5) clinically stable including vital signs stable and not vented or on continuous positive airway pressure; and (6) able to read and write Chinese at the fifth grade level.

We excluded patients with (1) major uncorrected hearing impairment, (2) major psychiatric illness (e.g., schizophrenia), (3) major uncorrected visual impairment, and (4) diagnosis of dementia.

### 3.3. Procedure

Participants were identified by reviewing the daily admission sheets and then were contacted as potential participants by the primary research investigator within 72 h after admission. Prior to participation in the study, each participant received a written consent form with information regarding the purpose, the content, and the significance of the study. Participants were informed about the time, the location, and the duration of data collection, as well as the possible benefits and risks of their participation. After clarifying the above-mentioned components and answering the participants’ questions, patients who agreed to take part in the study were asked to sign the consent form. After the informed consent, data from medical records and the participant were collected by the primary investigator. This procedure was continued to complete the sample size. In order to standardize the procedure, the primary investigator read the questions out loud to all participants. All questions were answered by the participants themselves. 

### 3.4. Variables and Instruments

The sociodemographic questionnaire: The demographic profile tool was researcher developed. Participants’ sociodemographic data were collected using a structured questionnaire. The following variables were included: age, gender, ethnicity, marital status, educational level, living arrangements, perceived economic status to meet needs, and religious affiliation.

Basic clinical information: This part included the following information: duration of heart failure (collect in months), NYHA functional class, recent LVEF (patients with LVEF less than 40% were also included), NT-proBNP, duration of symptoms (from time of onset to arrival at the hospital, collected in hours), and comorbid conditions, which were evaluated by the Charlson Comorbidity Index (CCI). NYHA functional class was routinely documented by physicians and was confirmed by the primary investigator who was trained to assess self-reported HF symptoms and limitations. Duration of heart failure and duration of symptoms was retrieved by interviews and confirmed by medical records. NYHA functional class, recent LVEF, and NT-proBNP were reviewed in the medical record. CCI is a world-wide tool which is used to assess the 10-year survival/mortality risk in patients with several co-morbidities [18].

Cognitive function: The Montreal Cognitive Assessment (MoCA) is a brief cognitive screening tool that can better detect mild cognitive impairment [19]. The Changsha version of MoCA demonstrated good reliability and validity [20]. It consists of eight cognitive fields: visual space and executive function, naming, memory, attention, language, concentration, working memory, and orientation to time and place. Because the average educational level of mainland Chinese is lower than that of Canadians, one point would be added additionally to the total score of the participants who were educated for less than 6 years when using the Changsha version of MoCA [20]. It took about 10 min to complete the questionnaire. Total score was 30, and a score below 27 points was considered as cognitive impairment. As many studies have reported that patients with HF are always accompanied with impaired cognitive function, patients were included in this study if they were alert and able to understand the data collection process and provided written informed consent.

Depression: The Patient Health Questionnaire (PHQ-8) was used to quickly assess depressive status in this study. The Patient Health Questionnaire eight-item depression scale consisted of eight items from the Diagnostic and Statistical Manual of Mental Disorders, fourth edition (DSM-IV) [21]. There was evidence that PHQ-8 was more convenient than PHQ-9 to use to identify patients with a current depression symptom, and a score ≥10 was considered as the cut-off point [22,23]. Each item was rated on a Likert scale from 0 (not at all) to 3 (nearly every day). Responses were summed to form a total score ranging from 0 to 24; higher scores indicated a higher level of depressive symptoms. Both the English version and the Chinese version of the PHQ-8 demonstrated good reliability and validity [23,24]. Scores ranging from 0 to 4 are considered as minimal depression, 5 to 9 as mild depression, 10 to 14 as moderate depression, 15–19 as moderately severe depression, and 20 to 24 as severe depression [21]. During the interview in this study, if a patient had a score of greater than or equal to 10, the primary investigator shared the result with his/her doctor.

Anxiety: The Patient-Reported Outcomes Measurement Information System (PROMIS) [25] Short Form v1.0–Anxiety 4a was used to measure anxiety in this study [26]. It can serve as an ultra-brief measure in many settings with good internal reliability [27]. Each item was scored from 1 to 5 on a Likert scale. Items within each domain were totaled, and raw scores were converted to standardized T-scores by PROMIS Assessment Center. Higher scores indicated more anxiety. 

Symptom perception: The Heart Failure Somatic Perception Scale v.3 (HFSPS) was an 18-item Likert scale used to assess perceived symptom burden of HF symptoms [28]. The HFSPS asked participants how much they were bothered by 18 common signs and symptoms in the past week using six response options ranging from 0 (I did not have the symptom) to 5 (extremely bothersome). Each item of HFSPS was calculated as a total score. Scores range from 0 to 90, with higher scores indicating higher perceived somatic awareness and symptom distress [29,30]. In addition to the total score, which has good reliability (Cronbach’s α: 0.90), a six-item dyspnea subscale, and seven-item early and subtle subscale were significantly associated with 1-year event-risk [29]. Psychometrics for the Chinese version of the HFSPS was 0.87 [31]. In this sample, the Cronbach’s α was 0.690. In the data collection process, this study was mainly interested in symptoms with daily activities, rather than at rest.

### 3.5. Data Analysis

Analyses were conducted using Epidata version 3.1 (The Epidata Association, Odense, Denmark) and IBM SPSS version 23.0 (SPSS Inc., Chicago, IL). All comparisons were two-tailed, and *p*-values equal to or less than 0.05 were considered as significant in this study. Clean up and analysis of missing data and outliers was performed before analyzing the data. Pearson (***r_p_***) and Spearman (***r_s_***) correlations were used to detect bivariate correlations of symptom perception and continuous variables. Correlations of symptom perception and binary variables was explored by using independent sample *t*-test (*t*) or non-parametric test (*t’*). Correlations of symptom perception and polytomous variables were explored by using single factor analysis of variance (*F*) or *Welch* test, and least significant difference (LSD) test was used to perform post hoc multiple comparisons. The α value was 0.05. To control the effects of covariates, all the demographic and clinical variables were analyzed in simple correlation analysis. Hierarchical multiple regression was used to further detect multivariable relationship of symptom perception and selected variables. For analysis, logarithmic transformation was used to normalize the distribution of NT-proBNP. For educational background of “bachelor or above”, perceived economic status to meet needs “less than enough”, NYHA functional class I, minimal depression was set as a reference category for each corresponding variable in hierarchical multiple regression.

## 4. Results

### 4.1. Description of the Characteristics of the Sample

A total of 285 hospitalized HF patients’ medical records were reviewed and invited, with 38 patients excluded (6 who were unable to understand the study, 10 who were clinically unstable, 2 with major uncorrected hearing impairment, 1 with major uncorrected visual impairment, and 19 with clinical data missing), and 39 patients refused to participate in the study. The remaining 208 patients completed the structured questionnaire with an effective rate of 100% (208/208). Table 1 describes the sociodemographic and clinical characteristics of the participants at baseline.

As shown in Table 2, the mean length of duration of heart failure was 40 months (range, 0.1 month to 324.4 months) and the length of duration of symptoms was over 2 weeks (426.29 ± 781.0). The mean LVEF was 38.23%, ranging from 20% to 65%. Most patients (60.1%) were labeled as heart failure patients with a reduced ejection fraction (HFrEF) [32]. The mean NT-proBNP of the participants was 8962.14 pg/mL, ranging from 759.0 to 64,372.0 pg/mL. A total of 12 participants had a normal NT-proBNP, and 196 patients had an abnormal level of NT-proBNP. Moreover, over half of the patients (76.9%) were at a high level of comorbidity status with a CCI score over 5. Most of the patients (51.0%) were at NYHA functional class III, and 65 (31.3%) at NYHA functional class IV. Nearly all the patients (98.6%) were cognitively impaired. Of the 208 patients, 22 (10.6%) patients scored over 10 on PHQ-8. Seventeen (8.2%) patients were at a high level of anxiety. 

The HFSPS was used to assess patients’ symptom perception ability. Table 3 displays the 18 items’ response of the participants in detail. Considering the purpose of this study, to explore the influencing factors of symptom perception, a total HFSPS score of each participant was also calculated. The HFSPS score was 43.16 ± 10.949, ranging from 7 to 69. Breathless (mean score was 3.217 ± 2.360, the alpha reliability was 0.765) and chest discomfort (the mean score was 2.952 ± 3.036, the alpha reliability was 0.374) were two symptoms that most reported by the participants. In the edema subscale, the mean score was 1.162 ± 2.358. The alpha reliability was 0.512. Either weight-gaining for the past week (82.7%) or swelling in the feet (39.4%) was ignored by most of the patients. As much as 63.5% of the sample reported they did not notice their shoes become tight at the end of the day. Scores of the early and subtle subscale were low (the mean score was 2.107 ± 2.053, the alpha reliability was 0.358). Over half of the patients (84.6%) reported that they did not feel clothes tight around their waist. Distress of stomach, reflected by the item “I feel uncomfortable in my stomach”, was reported by 44.2% of the patients. However, many participants (30.3%) said they were slightly distressed by losing their appetite.

### 4.2. Single Factor Analysis among Symptom Perception and Variables

The results of single factor analysis among symptom perception and variables are shown in Table 4. LVEF and NT-proBNP were statistically significant, whereas no statistical significance was found in age, HF duration, duration of symptoms, and MoCA score. Specifically, LVEF (*r*_s_ (208) = −0.221, *p* = 0.001) was negatively correlated with total HFSPS score. Meanwhile, NT-proBNP (*r*_p_ (208) = 0.226, *p* = 0.001) was positively correlated with total HFSPS score. The level of anxiety (*r*_s_ (208) = 0.179, *p* = 0.010) was also positively correlated with total HFSPS score. No significant correlation was found between gender, living arrangements, ethnicity, or religion and symptom perception in Chinese patients with heart failure. The differences in symptom perception among different educational levels (*Welch* (208) = 3.676, *p* = 0.023), different levels of perceiving economic status to meet needs (*F* (208) = 4.361, *p* = 0.014), different levels of depression (*F* (208) = 5.426, *p* = 0.001), and different NYHA functional classes (*F* (208) = 9.775, *p* = 0.000) were statistically significant and not identical. 

### 4.3. Hierarchical Multivariable Linear Regression Analysis Results

The final model was statistically significant (*F* = 4.335, *p* < 0.001), suggesting that there was a linear correlation between dependent variables and independent variables, and the inclusion of these four independent variables helped predict the dependent variables. Each included variable was independent of the other (Durbin–Watson test value, 1.559), and the variable tolerance was higher than 0.2 and variance inflation factor (VIF) was lower than 5, indicating that there was no multicollinearity in this study. In this model, 25.3% of the variation in symptom perception could be explained by NT-proBNP, level of anxiety, LVEF, educational level, perceived economic status to meet needs, and level of depression (*R*^2^ = 0.225, adjusted *R*^2^ = 0.173). Table 5 describes the results of the hierarchical multiple regression analysis of variables. 

Among the independent variables included in this study, the correlations between symptom perception and LVEF, educational level, NYHA functional class, and depression were statistically significant. The value of LVEF, the level of education, NYHA functional class, and depression were the main independent factors affecting the symptom perception ability of Chinese heart failure patients. Among them, LVEF was an independent risk factor for symptom perception ability. Symptom perception ability might decrease when the value of LVEF increases (B = −0.137, *p* = 0.026). Patients with primary school/below educational background, compared with bachelor or above educational background, had a significantly higher symptom perception ability (B = 8.926, *p* = 0.018). There was no significant difference in symptom perception among middle school, high school, and bachelor or above. Symptom perception of patients with NYHA functional class III and NYHA functional class IV was significantly higher than with NYHA functional class II. Moreover, symptom perception of patients with NYHA functional class IV was 6.723 times higher than with NYHA functional class II (B = 6.723, *p* = 0.003), and symptom perception of patients with NYHA functional class III was 4.313 times higher than with NYHA functional class II (B = 4.313, *p* = 0.032). The symptom perception of patients with mild depression was significantly higher than that of minimal depression (B = 3.563, *p* = 0.030). There was no significant difference in symptom perception between moderate depression and minimal depression, moderate severe depression, and minimal depression.

## 5. Discussion

To our knowledge, this is the first study to report the symptom perception in Chinese patients with heart failure. The results of this study support the Theory of Heart Failure Self-Care, Revised and Updated, that, as a key point in self-care, patients’ symptom perception might be conscious or subconscious and is driven by the interactions of the person, problem, and environmental factors. This study found that higher level of depression and the NYHA functional class were independent facilitators of symptom perception in Chinese patients with HF. However, higher level of the LVEF and educational level were independent risk factors of symptom perception ability.

The mean HFSPS score in this study was higher than similar studies in the United States and Italy, along with another study that used this scale for a different purpose in China [12,31,33,34,35]. In addition, the HFSPS was used to examine patients’ physical symptoms in China as well [31]. However, a detailed analysis of subscale items was not discussed. The following paragraphs review results of each sub-scale of the HFSPS.

*Chest discomfort.* Participants in this study reported a higher level of chest discomfort than those in prior studies [29,30,33]. Chest pain was experienced by 41% of patients in Jurgens’ (2006) study and was reported by 85.1% of the sample in this study. This might relate to the fact that most people in this sample were less educated and in a lower economic situation, especially the retired older adults. They lived in apartments without elevators and walked or took the bus to places they wanted to go to. Thus, they got more exercise in daily activities, especially after discharge, than patients in developed countries. This reason also explained the difference in the HFSPS total scores compared to prior studies.

*Dyspnea.* All patients reported dyspnea-related symptoms and rated themselves at a high level of distress. This finding was consistent with previous studies that also reported high rates and high distress of dyspnea [29,33]. However, there are also studies indicating that most of the patients did not report dyspnea or were less affected by these dyspnea-related symptoms [12,30]. Moreover, it is notable that “I woke up during the night because I had difficulty breathing” and “I was having difficulty breathing when putting on clothes” were always at a lower rating score in most of the prior studies, as was the same in this study. This might be because patients chose to utilize coping strategies, such as using a higher pillow, changing positions, or taking a break during dressing up, in order to alleviate shortness of breath during sleeping and getting dressed.

*Subtle symptoms*. Similar results were reported on stomach discomfort, cough, feeling tired, increased girth, needing more rest, and losing appetite [29,30,33,34]. Nevertheless, 84.6% of patients in this sample ignored increased girth, which was higher than in other prior studies. Patients wore loose clothes, as participants stated, which hampered their perception of waist circumference changes.

*Edema.* Similar to prior studies, less than half of the participants reported tight shoes or weight gain [29,33,34]. However, the number of patients who did not report tight shoes or weight gaining in this study was higher than other studies. These might relate to the cultural background of different countries. Most of the patients did not have the awareness of weight monitoring and did not use the weight scale at home. Even though the nurses proceeded to offer health education during hospitalization, patients did not go to the nurse station to measure their weight every day. Meanwhile, it is routine in China to remove shoes at the door and wear flip-flops or slippers in the home. Most elderly people liked to wear slippers or shoes that were more comfortable and softer, and thus they did not notice if the shoes became tight or not when edema occurred.

Clinical characteristics, including LVEF and NYHA functional class, were independently associated with symptom perception. Similar results had been found, and higher level of NYHA functional class predicted stronger ability of identifying dyspnea-related symptoms [36,37]. Meanwhile, as reported in prior studies, the higher NYHA functional class level and LVEF level indicated that more symptoms were experienced [38,39]. Symptom clusters, even in different cultural groups, were found to be significantly related to symptom perception [35,40,41]. A study reported that symptom education could significantly improve patients’ short-term prognosis [42]. Moreover, higher NYHA functional class was identified as a predictor of patients delay in seeking healthcare [34,43]. It is worth noting, however, that another study indicated that the presence and severity of signs and symptoms of heart failure were independent of LVEF values [44]. Therefore, future studies are recommended to enrolling patients with different LVEF types of heart failure with larger sample size, in order to further clarify the relationship between symptom perception ability and LVEF. Studies exploring whether the number of symptoms plays a mediating effect in the relationship between LVEF and symptom perception ability are also recommended. Clinicians need to recognize the importance of clinical characteristics including LVEF and NYHA functional class in HF.

Notably, the only sociodemographic characteristic independently associated with symptom perception was the level of education; patients with primary school/below educational background had a significantly higher symptom perception ability compared with bachelor or above educational background. This might be because participants with bachelor or above educational level were employed and some patients who were highly educated in this study were rehired after their retirement. They may not have had enough time and energy to sense clues of their health conditions [45]. However, a descriptive correlational study among 120 Egyptian heart failure patients found that college-educated patients had a better symptom perception ability than those with less education, a finding contrasting the results of our study [36]. Although significant differences were identified in implementing and evaluating treatment among different educational status groups, there were no differences on recognizing and evaluating change, which was the main variable in the current study. Other studies found no correlations between educational status and symptom perception [34,35,38,46]. Further studies exploring symptom perception with adequate samples of different educational status are recommended.

The final independent factor predictive of symptom perception was level of depression. It was interesting to note that symptom perception of patients with mild depression was significantly higher than that of minimal depression, presumably because patients with mild depression may be more likely to focus on their inner feelings and changes of the body. Again, findings of other studies vary in this regard. Some studies reported similar findings [47], whereas others found that depression made symptom perception difficult [48,49].

There was no significant relationship found between symptom perception and duration of symptoms, which might be explained by the high rates of cognitive impairment, where cognitive deficits hindered patients’ self-care ability [12]. Cognitive deficit is prevalent among individuals with heart failure, leading to higher risks of rehospitalization and dementia in patients with HF [50,51]. This discrepancy might be explained by the effect of educational level on cognition function and cognition assessment [52]. According to the Development Report on the Quality of Life for the Elderly in China [53], the educational level of the elderly in China is generally low, and about 71.1% have had only a primary school educational background or never went to school, a finding replicated in this study.

## 6. Limitations

This study is the first study to preliminarily explore symptom perception in Chinese patients with heart failure and expands the understanding of symptom perception influencing factors in HF. The applicability of the Theory of Heart Failure Self-Care has been demonstrated, and it is recommended for use as theoretical guidance for intervention studies and other studies in the future. However, the generalizability of the results of this study is limited due to a moderate sample size with limited diversity in ethnicity and religious affiliation. The nature of the sample should be considered when comparing the results to other populations. In addition, because all the questions in this study were read to the participants by the research investigator where the participants needed to speak out their answers by themselves in the process, the answers might have been given after careful consideration, resulting in subjective evaluation bias of the participants. Finally, as majority of the patients in this sample had cognitive impairment, there might be recall bias.

## 7. Conclusions

Symptom perception plays an important role in the self-care of patients with heart failure. The symptoms of each heart failure patient might be similar, but the ability to perceive changes in symptoms differs. Understanding what patients with heart failure perceive symptom changes to be can help to build up a better healthcare among this population. This study found that level of depression, the NYHA functional class, the LVEF, and educational status were the independent predictors of symptom perception abilities. At the same time, this study suggests that we might be able to identify patients with poor symptom perception by using these indicators in the future. It helps to clarify whether the patients can perceive the changes of symptoms and to help healthcare providers provide more personalized care and targeting interventions that are more consistent with the patient’s situation.

Moreover, this study emphasizes that health policy and healthcare providers should establish closer contact with heart failure patients with depression to help them eliminate these problems. In China, more health policies that focus on patients with heart failure should be developed and implemented. These recommendations are also recommended for use in the health management of community-based patients with heart failure.

## Figures and Tables

**Table 1:** Sociodemographic characteristics of the sample (*N* = 208).

Variable	Mean ± SD (Range)	*N* (Percentage)
Age (years)	67.75 ± 11.9 (33–93)	-
Male	-	124 (59.6)
Ethnicity		
Han nationality	-	202 (97.1)
Minority	-	6 (2.9)
Education		
Primary school or below	-	113 (54.3)
Middle school	-	51 (24.5)
High school	-	36 (17.3)
Bachelor or above	-	8 (3.8)
Marital status		
Married	-	168 (80.8)
Single	-	4 (1.9)
Divorced or separated	-	0 (0)
Widowed	-	36 (17.3)
Living arrangements		
Living alone	-	25 (12.0)
Living with others	-	183 (88.0)
Perceived economic status to meet needs		
Less than enough	-	41 (19.7)
Enough	-	164 (78.8)
More than enough	-	3 (1.4)
Religious affiliation		
No	-	199 (95.7)
Yes	-	9 (4.3)

**Table 2:** Description of the clinical characteristics of the study participants (*N* = 208).

Variable	Mean ± SD (Range)	*N* (Percentage)
Duration of symptoms (hours)	426.29 ± 781.0 (8–8640)	
Duration of heart failure (months)	40.26 ± 51.0 (0.1–324.4)	
NT-proBNP (pg/mL)	8962.14 ± 11,262.2 (759.0–64,372.0)	
LVEF (%)	38.23 ± 12.2 (20–65)	
< 40%		125 (60.1)
40%–49%		36 (17.3)
≧50%		47 (22.6)
CCI score (comorbidity status)	5.793 ± 1.939 (1–11)	
1–2		9 (4.3)
3–4		39 (18.8)
≥5		160 (76.9)
NYHA functional class:		
I		0
II		36 (17.3)
III		107 (51.4)
IV		65 (31.3)
PHQ-8 score (depression):	4.279 ± 4.529 (0–17)	
0–4		107 (51.4)
5–9		79 (38.0)
10–14		16 (7.7)
15–19		6 (2.9)
20–24		0
PROMIS T-score (anxiety):	6.418 ± 2.817 (4–18)	
<60		191 (91.8)
>60		17 (8.2)
MoCA score (cognitive status):	17.904 ± 4.482 (8–30)	
≤26		205 (98.6)
>27		3 (1.4)

LVEF, left ventricular ejection fraction; CCI, Charlson Comorbidity Index; NYHA, New York Heart Association; PHQ, Patient Health Questionnaire; PROMIS, Patient-Reported Outcomes Measurement Information System; MoCA, Montreal Cognitive Assessment.

**Table 3:** Item responses of Heart Failure Somatic Perception Scale (*N* = 208).

Items	I Did not Have This Symptom	Did not Bother Me at All	Slight Distress	Moderate Distress	Strong Distress	Severe Distress	Mean ± SD	Skewness	Kurtosis
Chest discomfort									
I can feel the heartbeat getting faster (item 1)	21.6	2.9	12.5	19.7	20.2	23.1	2.83 ± 1.817	−0.421	−1.166
I feel discomfort or pain in chest (item 3)	14.9	4.8	12.0	13.5	36.1	18.8	3.07 ± 1.665	−0.718	−0.732
***Dyspnea***									
I cannot breathe when lying down (item 2)	11.1	1.4	9.1	21.2	23.1	34.1	3.46 ± 1.594	−0.960	−0.035
I feel like I cannot catch my breath (item 7)	6.3	1.0	4.8	11.5	39.9	36.5	3.87 ± 1.324	−1.635	2.344
I woke up during the night because I had difficulty breathing (item 9)	33.2	2.4	9.1	17.8	18.3	19.2	2.43 ± 1.955	−0.117	−1.543
I cannot perform normal activity because of shortness of breath (item 12)	6.7	1.4	2.4	13.9	43.8	31.7	3.82 ± 1.317	−1.670	2.507
I was having difficulty breathing when putting on clothes (item 13)	41.3	5.3	17.3	18.8	12.0	5.3	1.71 ± 1.667	0.367	−1.223
It is difficult for me to breathe (item 17)	5.3	.5	3.8	8.7	41.8	39.9	4.01 ± 1.239	−1.894	3.603
Early and subtle									
I feel uncomfortable in my stomach (item 4)	44.2	1.4	22.6	17.3	7.7	6.7	1.63 ± 1.657	0.477	−1.008
I had a cough (item 5)	40.9	1.9	28.4	4.3	13.0	11.5	1.81 ± 1.799	0.475	−1.128
I feel tired (item 6)	5.3	1.4	4.8	12.0	50.0	26.4	3.79 ± 1.232	−1.666	2.766
I feel my clothes are tight around my waist (item 14)	84.6	3.4	6.3	1.9	2.4	1.4	0.38 ± 1.029	2.905	8.023
I woke up at night because I had to urinate (item 15)	12.0	27.4	20.7	24.0	12.5	3.4	2.08 ± 1.342	0.210	−0.828
I need more rest during the day than normal (item 16)	7.7	9.1	16.8	24.0	32.2	10.1	2.94 ± 1.399	−0.559	−0.505
I do not have an appetite (item 18)	28.8	8.7	30.3	18.3	10.6	3.4	1.83 ± 1.46	0.202	−0.912
Edema									
My feet are swollen at the end of the day (item 8)	39.4	1.4	16.3	9.1	13.5	20.2	2.16 ± 2.008	0.186	−1.560
I feel my shoes become tight at the end of the day (item 10)	63.5	7.7	12.0	6.7	4.8	5.3	0.98 ± 1.521	1.405	0.768
I gained weight over the past week (item 11)	82.7	5.3	8.7	1.9	1.0	0.5	0.35 ± 0.854	2.742	7.815

**Table 4:** Single factor analysis among symptom perception and variables (*N* = 208).

Variable	*r_p_*/*r_s_*	*t*/*t’*	*F*/*Welch*	*p*
Age	−0.045			0.521
HF duration	0.041			0.555
LVEF	−0.221			0.001 *
NT-proBNP	0.226			0.001 *
Duration of symptoms	−0.079			0.257
MoCA	−0.025			0.724
Levels of anxiety	0.179			0.010 *
Gender		−0.454		0.650
Living arrangements		0.679		0.498
Ethnicity		−1.137		0.257
Religion		−0.139		0.890
Educational background			3.676	0.023 *
Marital status			1.419	0.244
Perceived economic status to meet needs			4.361	0.014 *
Levels of depression			5.426	0.001 *
NYHA functional class			9.775	0.000 *
Comorbidity status			0.308	0.735

HF, heart failure; LVEF, left ventricular ejection fraction; MoCA, Montreal Cognitive Assessment; *r_p_*, Pearson correlation coefficient; *r_s_*, Spearman correlation coefficient; *t*, independent sample *t*-test; *e*, non-parametric test; * at the 0.05 level (two-tailed), the correlation was significant.

**Table 5:** Hierarchical multivariable regression analysis of symptom perception and variables (*N* = 208).

Variable	Unstandardized Coefficients		Standardized Coefficients	*t*	Significance	95.0% Confidence Interval for B	
	B	SE	β			Lower Bound	Upper Bound
NT-proBNP	1.660	1.743	0.067	0.953	0.342	−1.777	5.098
PROMIS	0.335	0.307	0.086	1.093	0.276	−0.269	0.940
LVEF	−0.137	0.061	−0.152	−2.241	0.026 *	−0.257	−0.016
Education (primary school/below)	8.926	3.754	0.407	2.378	0.018 *	1.522	16.329
Education (middle school)	6.403	3.912	0.252	1.637	0.103	−1.312	14.118
Education (high school)	7.431	3.994	0.257	1.86	0.064	−0.447	15.309
Meet needs (enough)	−3.549	1.812	−0.133	−1.959	0.052	−7.123	0.024
Meet needs (more than enough)	−3.657	6.228	−0.04	−0.587	0.558	−15.94	8.626
NYHA functional class III	4.313	1.998	0.197	2.159	0.032 *	0.373	8.253
NYHA functional class IV	6.723	2.239	0.285	3.002	0.003 *	2.306	11.139
Mild depression	3.563	1.634	0.158	2.181	0.030 *	0.341	6.785
Moderate depression	4.542	2.89	0.111	1.572	0.118	−1.157	10.242
Moderately severe depression	2.854	4.847	0.044	0.589	0.557	−6.705	12.413

* At the 0.05 level (two-tailed), the correlation was significant.
